# The effect of formal fetal movement counting on maternal psychological outcomes: A systematic review and meta-analysis

**DOI:** 10.18332/ejm/145789

**Published:** 2021-02-03

**Authors:** Nazia AlAmri, Valerie Smith

**Affiliations:** 1 King Hamad University Hospital, Busaiteen, Bahrain; 2 School of Nursing and Midwifery, Trinity College Dublin, Dublin, Ireland

**Keywords:** fetal movement assessment, maternal–fetal attachment

## Abstract

**INTRODUCTION:**

Formally counting fetal movements in pregnancy is one of the oldest methods to assess fetal well-being. Although not routinely recommended in contemporary maternity care, due to a lack of evidence of its effectiveness, formal fetal movement counting is still practiced in many birth settings. Requesting women to formally count their fetal movements in a structured, objective way that can potentially improve maternal subjective outcomes such as worry or concern. The aim of this study was to evaluate the effect of formal fetal movement counting versus no formal counting, on maternal worry, concern or anxiety, and maternal–fetal attachment (MFA). Secondary outcomes were compliance with the intervention (counting method) and hospital admission/attendance for fetal activity concerns.

**METHODS:**

CINAHL, MEDLINE and EMBASE were searched systematically for eligible studies from inception dates to June 2020, supplemented by searches of trial databases, grey literature and the reference lists of included studies. Randomized controlled and quasi-randomized trials were included in the review.

**RESULTS:**

Nine studies reported across 15 publications were included involving 70824 pregnant women. The results showed that MFA levels were significantly higher in women who formally counted fetal movements than those who did not (standardized mean difference=0.72; 95% CI: 0.10–1.33, five studies, 1565 women). There were no differences between the groups in maternal anxiety or worry/concern outcomes. Attendance or admission rates for reduced fetal movements, or concern for fetal activity, did not differ between the groups (OR=1.36; 95% CI: 0.97–1.91, three studies, 1947 women). Compliance in completing fetal movement charts varied, ranging 45–90%, although definitions of compliance differed across studies, which may have affected rates.

**CONCLUSIONS:**

This review has found that formal fetal movement counting in pregnancy has no detrimental effects on maternal psychological or emotional status and positively affects maternal–fetal attachment. Although current evidence does not support the use of formal fetal movement counting for improving perinatal outcomes, such as stillbirth and neonatal death, the results of this review are helpful for clinicians in discussing fetal movements in pregnancy and in discussing the optional methods available to women who may be advised to or choose to objectively assess fetal movements using a formal fetal movement counting method.

## INTRODUCTION

Quickening describes the first fetal movements (FMs) felt by a woman and is a presumptive sign of pregnancy. These first FMs are defined by a kick, flutter, or roll^1^, and are first experienced by women generally between 16 and 22 gestational weeks. Formal fetal movement counting (FMC) was introduced in the 1960s to objectively assess FMs. FMC practice generally adopts the use of a ‘kick’ or FM chart on which a woman records the times and numbers of FMs felt over the course of the day^2^. Other more technical methods such as Multisensory Magnetocardiographic Recordings (MMR), mobile applications and abdominal sensors have also been developed for objectively assessing FMs in pregnancy and have been used more recently^3-5^. However, evidence supporting formal FMC for improving maternal and neonatal clinical outcomes is lacking. A recent systematic review and meta-analysis of randomized controlled trials that compared perinatal outcomes in women who were instructed to formally count their FMs (n=262059) and women who received standard antenatal care (n=196542), for example, found no differences between the groups in the outcomes of stillbirth, neonatal death, small for gestational age or admission to the neonatal intensive care unit^6^. Furthermore, slight statistically, but non-clinically, significant increases in the rates of induction of labor and caesarean section were observed in the formal FMC group^6^. In line with current evidence, formal FMC is not routinely recommended in contemporary maternity care^7,8^, yet contrary to this, the objective assessment of FMs using formal FMC remains a practice in some birth settings, in particular, for women with high-risk pregnancies^9,10^.

Given this evidence-practice gap, consideration of the effect of formal FMC beyond clinical outcomes is warranted, as FMC has the potential to also affect maternal psychological states, either positively or negatively. The use of kick charts, for example, have been associated with higher levels of maternal–fetal attachment (MFA) and enhanced communication with the fetus^1,11,12^. Others, however, have reportedly found no difference in MFA when women use objective FMC compared to no FMC, suggesting instead that levels of MFA are affected by other influencing factors such as women approaching the end of their pregnancy or possible fear of childbirth^13^. Furthermore, structured assessment of FMs may result in increased antenatal visits related to maternal concerns for FMs^2,14,15^, or conversely, a reduction in the number of unscheduled visits because women become more aware of fetal activity and the norms for their baby^16^. Studies have also found, however, that up to as many as 50% of pregnant women do not receive information about FMs from their healthcare providers^17,18^, and, as a result, women do not understand the importance of being aware of their FMs in pregnancy^2^. Understanding the impact or effects of formal FMC on women’s subjective outcomes such as concern/worry and anxiety is essential for healthcare providers who may guide women on assessing or being aware of their FMs in pregnancy. Knowing the potential for positive or negative psychological effects resulting from formal FMC allows healthcare providers to discuss such possible effects with women as part of an overall approach in discussing FMs in pregnancy. For this reason, we conducted a systematic review and meta-analysis of the impact of formal FMC on the measures of maternal worry, concern or anxiety and maternal– fetal attachment, with the aim to evaluate the impact of formal fetal movement counting compared to no formal counting on subjective maternal psychological outcomes. The Preferred Reporting of Items for Meta-Analyses (PRISMA) Checklist was used in reporting the systematic review (http://prisma-statement.org/prismastatement/Checklist.aspx).

## METHODS

The population, intervention, comparator, and outcomes (PICO) format was used to define the review’s inclusion criteria (Table 1). Randomized controlled trials (RCTs) (including cluster or crossover trials) and controlled or quasi-randomized trials were eligible for inclusion.

**Table 1:** Inclusion criteria

P	Pregnant women of any parity greater than 20 weeks gestation with high- or low-risk pregnancies. The 20-week gestational age cut-off was chosen as most pregnant women experience FMs by this time.
I	Any method of formally counting FMs in pregnancy. This may include the use of fetal movement or kick-charts, FM monitoring devices, or other structured approaches that involve counting FMs in pregnancy.
C	No formal counting strategy.
O	Maternal subjective outcomes, although these may have been measured objectively using validated tools (e.g. Cambridge Worry Scale)

### Search and selection strategy

A comprehensive search of the electronic sources was implemented. No limitations on language or date were applied in the searches. The databases of CINAHL, MEDLINE and Embase were searched from their year of inception to March 2020 and updated again in June 2020. The search terms were developed around the concept of FM and included key terms and Medical Subject Headings (MeSH). Terms used were: Fetal Movement* OR Fetal Activity OR Fetal Kick* OR pregnancy kick* OR Fetus movement* OR Fetal monitor* OR Fetal wellbeing OR Fetal well-being OR Fetal movement count* OR Fetus movement* OR Fetal kick count*. Grey literature online sources were also searched (www.opengrey.org) to identify any further potential trials of relevance to the review. Trial registries were screened for any ongoing trials. These included the World Health Organization (WHO) International Clinical Trials Registry Platform (ICTRP), the International Standard Randomized Controlled Trials Number (ISRCTN) registry and clinicaltrials.gov. Conference proceedings of the International Confederation of Midwives (ICM) Triennial Conference (2017) were also screened for additional studies that might not have been captured by the electronic search. Lastly, the reference list of each included study was screened for possible additional studies that could be included.

### Outcomes

The primary outcomes of interest to the review were: 1) maternal anxiety or concern/worry (as measured using a validated scale or as reported by the study authors); and 2) maternal–fetal attachment or bonding (as measured using a validated scale or as reported by the study authors). The secondary outcomes of interest were: 3) maternal adherence/compliance to the intervention; and 4) visits to the clinic/hospital for reduced FM or any concern regarding fetal activity.

### Risk of bias/quality assessment

The quality assessment tool for quantitative studies, developed by Effective Public Health Practice Project (EPHPP) (https://www.ephpp.ca/quality-assessment-tool-for-quantitative-studies/) was used to assess the methodological quality of the included studies. The tool was chosen as an appropriate tool because it allows for the assessment of the quality of both RCT and non-RCT designs. The tool consists of eight categories: 1) selection bias, 2) study design, 3) confounders, 4) blinding, 5) data collection method, 6) withdrawals and dropouts, 7) intervention integrity, and 8) analysis. Once each category is assessed, an overall judgement as to each included study’s quality, based on strong, moderate, or weak judgements, was made. The quality assessment was conducted by NA and corroborated by VS. Any discrepancies that emerged were discussed and resolved by consensus.

### Data extraction

A pre-designed data extraction form was used to extract relevant data from the included studies. The information extracted included: the aim of the study, location and study setting, year study was done, sample size and description of the study population, description of the intervention and comparator, and results related to the review’s pre-specified outcomes.

### Data analysis

Dichotomous data were analyzed using odds ratios and 95% confidence intervals (CI). The summary effect measure for continuous data was the mean difference, where the same outcome was measured across the included studies in the same way. Where the same outcome was measured differently across the studies (e.g. using different scales or measurement tools), the standardized mean difference (SMD) was used for the meta-analysis. A fixed effects model was used for meta-analysis unless there was moderate or high heterogeneity as defined by an I2 >50%^19^. In such analyses, a random effects model was applied. The numerical data from each study for each outcome were included in a meta-analysis, where possible, using Review Manager (RevMan) version 5.3. When it was not possible to combine the studies’ results in a meta-analysis, the results are reported narratively.

## RESULTS

### Results of search and selection strategy

**Figure 1:**
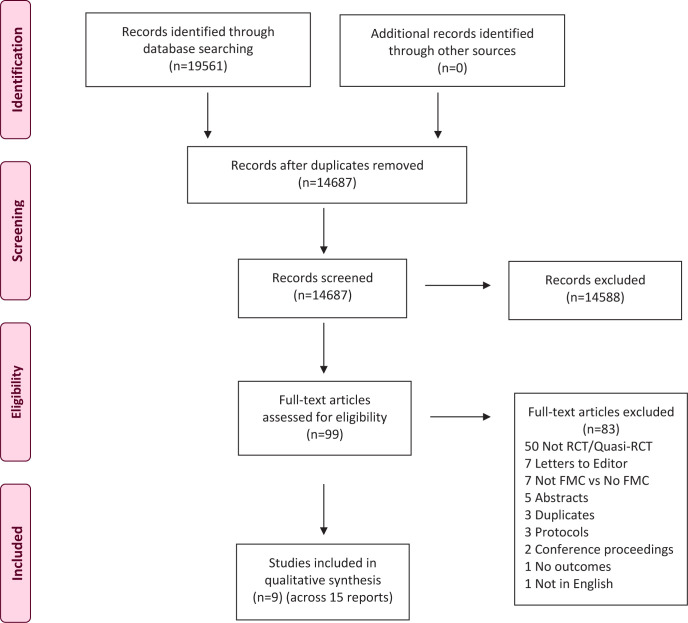
Search and selection process

The search of the electronic databases yielded 19561 citations. Of these, 4874 were identified as duplicates and were removed. The remaining 14687 were screened on title and abstract of which 14588 were excluded as they were clearly not eligible. Full texts of the 99 remaining citations were retrieved, and further screened for eligibility. Of these, 83 were excluded as 50 were not RCT or quasi-RCT designs; 7 were commentaries or letters to the Editor; 7 did not involve a comparison of formal FMC versus no FMC; in 5 the abstract or full text were not available and so their eligibility could not be fully assessed; 3 were protocols or ongoing studies; 3 were further duplicate reports; 2 were conference proceedings/abstracts that did not have sufficient information to include them; 1 did not measure any of the review’s pre-specified outcomes; and 1 was a non-English language publication. On further review of one included study, we found that formal FMC was not part of the intervention, rather women were instructed to monitoring the character, strength, and frequency of FMs, but not to count each FM. We subsequently excluded this study^16^. The search resulted in 9 eligible studies, reported across 15 publications which were included in the review^13,14,20-32^. Figure 1 illustrates the search and selection process.

### Description of included studies

Table 2 presents the characteristics of the included studies. A total of 70824 women were involved in the 9 studies. Six studies were RCTs of which one was a cluster RCT27. In two other studies (three reports) the authors note random allocation, but the method was not described20,21,26. The remaining study was a non-randomized trial32. In all nine studies, FMC was compared to standard antenatal care involving no formal FMC. Table 3 illustrates the review’s pre-specified outcomes that were reported in each study. Table 4 describes the instruments, where relevant, that were used to measure the outcomes.

**Table 2:** Characteristics of included studies

*Reference*	*Aim of study*	*Design*	*Study setting*	*Participants*	*Intervention*	Control*
Âbasi et al.^20,21^	To determine the effect of FMC on MFA	Random allocation (method unknown)	Six healthcare centers, Sari, Iran	Low-risk pregnancy, primigravid, 18–35 years, at least primary school education and gestational age 28–32 weeks, and having no obstetric or psychological problems.	FMC every morning after breakfast for one month N=42	Standard ANC N=41
Delaram et al.^22-25^	To determine the effect of FMC on pregnancy outcomes	RCT	Two health centers in Iran	Nulliparous women with singleton pregnancy, 28–37 gestational weeks, no history of the mental illness referred to Health Centers Oct 2012 – Dec 2013.	FMC every morning for 30 min from 28–37 weeks N=100	Standard ANC N=108
Gibby^26^	To compare maternal anxiety in a low-risk pregnant population between women who kept daily FM charts and women who did not	Random allocation (method unknown)	Prenatal clinic, Florida, US	Low-risk pregnant population, 33–37 gestational weeks, mean age 23 years	Daily FMC; Cardiff count-to-ten chart N=16	Standard ANC N=17
Grant et al.^27^	To examine if formal FMC backed by appropriate action reduced antepartum fetal death	Cluster RCT	UK, Belgium, Sweden, Ireland, and USA	The clusters (n=26, approx. 1000 women in each) consisted of all women who would be receiving maternity care from an obstetrician, clinic, or hospital during recruitment, 28–32 weeks gestation.	Daily FMC; Cardiff count-to-ten chart N=31993	Standard ANC N=36661
Guney and Ugar^28^	To determine the effect of FMC on MFA	RCT	Six family health centers, Malatya Province, Turkey	Low-risk singleton pregnancy, 28–32 weeks gestation.	Daily FMC; Count-to-ten method N=55	Standard ANC N=55
Liston et al.^29^	To ask whether mothers would monitor fetal activity, and if they did, whether such monitoring would cause deleterious psychological effects	RCT	Women were referred to the study from 15 Family Physician offices, Canada	Low-risk primigravida, aged 19–35 years, 28–37 gestational weeks, with no pre-existing medical or psychological problems. Jan 1986 – Jun 1988.	Daily FMC; Modified Cardiff count-to-ten N=178	Standard ANC N=195
Mikhail et al.^30^	To examine the effect of FMC on MFA	RCT	Prenatal clinic, Bronx Municipal Hospital Center, New York	Uncomplicated singleton pregnancies, gestational age 28–32 weeks.	Sadovsky method N=63 Cardiff method N=62	Standard ANC N=88
Saastad et al.^13,14,31^	To examine the effects of FMC on perinatal outcomes, MFA levels and maternal concern	RCT	Nine Hospitals, Norway	Singleton pregnancies, excluding pregnancies with severe anomalies or other causes for considering termination of the pregnancy, Sept 2007 – Nov 2009.	Daily FMC from 28 weeks N=554	Standard ANC N=532
Singh and Sidhu^32^	To examine if daily FMC charts would reduce perinatal mortality in low-risk pregnancy	Non-randomized trial	Military Hospital, India	Pregnant women in their ninth month of pregnancy.	FMC for one hour daily after food; breakfast, lunch, or dinner N=250	Standard ANC N=250

ANC: antenatal care. FM: fetal movements. FMC: Fetal movement counting. MFA: maternal–fetal attachment. RCT: randomized control trial.

* The control group in all studies involved standard ANC with no formal FMC.

**Table 3:** Pre-specified outcomes reported in each study

*Study*	*Anxiety*	*Worry/Concern*	*MFA*	*Compliance to FMC*	*Hospital attendance*
Âbasi et al.^20,21^			+		
Delaram et al.^22-25^	+	+	+		+
Gibby^26^	+				
Grant et al.^27^	+			+	+
Güney and Uçar^28^			+		
Liston et al.^29^	+	+	+	+	+
Mikhail et al.^30^			+		
Saastad et al.^13,14,31^		+	+	+	+
Singh and Sidhu^32^					+

**Table 4:** Instruments used to measure outcomes

*Scale*	*Description*	*Direction*	*Studies using scale*
Spielberger Trait and Anxiety Inventory (STAI) Scale	A self-report questionnaire with subscales that measure trait and state anxiety. Items are rated on a 4-point scale (1 to 4) from ‘almost never’ to ‘almost always’. Min score is 20 and max is 80.	Higher scores indicate greater levels of anxiety	Delaram et al.^23^ Gibby^26^ Liston et al.^29^
Cambridge Worry Scale (CWS)	16-item instrument measuring women’s major worries during pregnancy. Responses are made on a 6-point (0 to 5) Likert-type scale ranging from ‘not a worry’ to ‘major worry’.	Higher scores indicate greater levels of worry	Saastad et al.^14^
The Prenatal Attachment Inventory (PAI)	A 21-item inventory measuring how often the mother has affectionate thoughts or behaves affectionately toward the fetus. Responses are rated on a 4-point (1 to 4) Likert-type scale; total score 21 to 84.	Higher scores indicate greater levels of attachment	Saastad et al.^13^ Delaram et al.^25^
Maternal Antenatal Attachment Scale (MAAS)	A 19-item, 5-point Likert-type scale is used for each item (5 represents ‘strong emotions toward the fetus’ and 1 represents ‘the absence of feelings toward the fetus’). The scale has two subdimensions: the quality of attachment (10 items, score 10–50) represents the quality of emotional experiences of a pregnant woman for the fetus; the amount of time spent in attachment (8 items, score 8–40) represents the intensity of pregnant women’s preoccupation with the fetus and thinking about, talking with, and touching the fetus.	Higher scores indicate greater levels of attachment	Güney and Uçar^28^
Maternal Fetal Attachment Scale: Cranley - 24 item	24-item, 5-point Likert-type scale describing baby-related thoughts and actions of expectant mothers. Responses are ‘most of the time, frequently, sometimes, rarely, never’, scored from 5 to 1.	Higher scores indicate greater levels of attachment	Âbasi et al.^20,21^ Mikhail et al.^30^
Maternal Attitudes toward Pregnancy Inventory (MAPI)	48 items, each item rated 1 to 4; overall scores range from 48 to 192. The scale contains four individual factors: feelings of well-being, pride in pregnancy, concerns for birth, and attitudes toward infant.	Higher scores indicate higher strength of attitude	Liston et al.^29^

### Methodological quality of included studies

Table 5 presents the global and component ratings for the methodological quality of the included studies. Three studies (four reports) were assessed as strong on methodological quality^20,21,27,30^, four as moderate (eight reports)^13,14,22-25,28,29,31^ and two as weak^26,32^. Regarding individual methodological quality components, two studies (five reports) were assessed as strong for selection bias with an 80–100% participation rate^15-22,29^. The remaining seven studies were assessed as moderate on this criterion as less than 80–100% of those invited took part, or because the information to accurately assess participation rates was missing from the study report. Eight studies used random allocation of participants and were assessed as strong on the study design component. The remaining study was rated moderate as it was a non-randomized trial^32^. Six studies considered characteristic variables to assess uniformity at baseline (e.g. age, education level, marital status, gestational age, outcome measures at baseline) and were assessed as strong on confounding. Two studies were assessed as weak due to the lack of information in the study report^26,32^. Due to the nature of the intervention, blinding of participants and personal information was not possible, although blinding of outcome assessors was possible; all nine studies were rated as either moderate or weak on blinding. Only one study did not specify the tool used to count FM and was thus assessed as weak for data collection methods^32^. Seven studies specified the percentage of withdrawals and dropouts as less than 80%; these studies were rated as strong on this component. One study was rated moderate as it had a 36% withdrawal and dropout rate^28^. The remaining study was assessed as weak due to a lack of information in the study report to assess this component^26^.

**Table 5:** Methodological quality of included studies

*Study*	*Global rating*	*Selection bias*	*Study design*	*Confounders*	*Blinding*	*Data collection methods*	*Withdrawals and dropouts*
Âbasi et al.^20,21^	**S**	M	S	S	M	S	S
Delaram et al.^22-25^	**M**	S	S	S	W	S	S
Gibby^26^	**W**	M	S	W	M	S	W
Grant et al.^27^	**S**	M	S	M	M	S	S
Güney and Uçar^28^	**M**	M	S	S	W	S	M
Liston et al.^29^	**M**	S	S	S	W	S	S
Mikhail et al.^30^	**S**	M	S	S	M	S	S
Saastad et al.^13,14,31^	**M**	M	S	S	W	S	S
Singh and Sidhu^32^	**W**	M	M	W	M	W	S

S: strong; M: moderate; W: weak.

## RESULTS

### Maternal anxiety and worry/concern

Four studies assessed the effect of FMC on maternal anxiety^23,26,27,29^. Three studies used the STAI scale to measure anxiety^23,26,29^. In the fourth study, it was not clear how anxiety was measured. Still, the authors reported that a slightly higher proportion of women in the counting group felt very or quite anxious in late pregnancy (difference in mean 2.0 per 100 women, 95% CI: -1.8 – 5.8). However, this difference was not significant^27^. Complete data for a meta-analysis were not available for the three remaining studies, and the results are reported narratively. Delaram and Shams^23^ reported anxiety separately by state and trait anxiety and found significantly lower levels of anxiety on both state and trait dimensions in the FMC group (state anxiety: MD=2.91; 95% CI: -5.58 – -0.24; trait anxiety: MD= -3.27; 95% CI: -5.64 – -0.90; 208 women). Liston^29^ conversely reported an increase in state anxiety from 32.24 and 30.77 at baseline to 31.84 and 32.15 (p=0.043) at the end of the study in the intervention and control groups, respectively. A decrease in trait anxiety, however, was found, from 34.82 and 33.65 at baseline to 33.19 and 34 .02, in the intervention and control groups, respectively (p=0.013). In the remaining study, the authors simply reported that there was no significant difference between the groups at the 0.05 level for the outcome anxiety^26^.

Maternal concern/worry was reported in three studies^14,24,29^. Liston et al.^29^ in reporting ‘concern for delivery’, reported group means which were similar for both groups (10.39 versus 10.45: intervention versus control). Maternal concern/worry was reported in different formats in the two other studies; the results indicated no differences in concern/worry between the FMC and standard care groups:

- Concern for fetal activity (MD=0.25; 95% CI: - 012 – 0.62; 208 women^24^ and OR=1.12; 95% CI: 0.87–1.44; 1013 women)^14^.
- Overall concern/worry using the Cambridge Worry Scale (MD= -0.13; 95% CI: -5.51–5.25; 1013 women)^14^.
- Use of the FMC chart alone caused 8% of participants to be concerned.

### Maternal–fetal attachment

**Figure 2:**
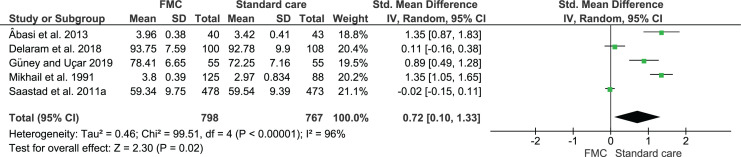
Maternal-fetal-attachment

MFA was reported in six included studies^13,20,21,25,28-30^. Five of the six studies contributed to meta-analysis. In one study, that of Liston et al.^29^, the group means only, without SDs, were provided, and these means appear similar (29.59 versus 29.37 for intervention and control groups, respectively)^29^. Data for the remaining five studies were included in the meta-analysis. As the studies used different scales to measure MFA (Table 4), the Standardized Mean Difference (SMD) was calculated. The results found that levels of MFA were significantly increased in women who formally counted FM compared to those receiving standard care (SMD=0.72; 95% CI: 0.10–1.33, five studies, 1565 women) (Figure 2). Due to high statistical heterogeneity, a random-effects model was applied, although heterogeneity remained high (96%).

In addition to the overall MFA scores, Güney and Uçar^28^ also explored the quality of MFA and time spent in MFA. The results found higher MFA quality and time spent in MFA with FMC compared to standard care (quality: MD=1.98; 95% CI: 0.75–3.21; time: MD=4.82, 95% CI: 3.19–6.45).

### Hospital attendance or admission

**Figure 3:**
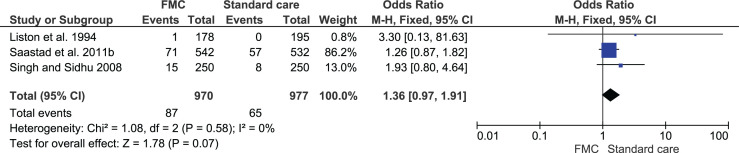
Hospital attendance

Clinic or hospital attendance or admission for concern regarding FMs was reported in five studies^13,23,27,29,32^. Three of the five studies contributed data to a meta-analysis (Figure 3). The analysis showed no difference in admission rates between the groups (OR=1.36; 95% CI: 0.97–1.91; 1947 women). The remaining two studies reported the outcome continuously and found no differences between the groups in hospital admission rates: MD=8 (-3 to 19)^27^ and 1.95 (SD=0.21) versus 1.93 (SD=0.21)^23^.

### Compliance with FMC

Compliance in completing FMC charts, regardless of FM counting method, was measured in three studies^13,27,29^, but only in women allocated to the intervention group in two studies^13,29^. For this reason, a meta-analysis was not performed, and we report the results narratively. Compliance in the study of Liston et al.^29^ was measured by >95% chart completion^29^. Saastad et al.^13^ defined compliance as a completed chart at least twice weekly and in more than 50% of the days. In the third study, the measure of compliance is not clear, but appears to be based on numbers completing FMC charts^27,33^. A total of 32350 women were allocated to FMC in the three studies. Of these, 14793 completed FMC charts as per compliance definitions, providing an overall compliance rate of 55%. The result, however, is heavily influenced by the Grant trial whereby FMC charts were available for only 45% of women in the counting cluster^27^. This compares with compliance rates of 90% and 85% in the other two studies, respectively.

## DISCUSSION

FMs in pregnancy are an important indicator of fetal health and can be associated with maternal subjective experiences. The results of this systematic review show that maternal psychological well-being is not adversely affected by formal FMC. Levels of MFA were significantly increased, including the overall quality and time spent in MFA, in women who formally count FMs. No differences between groups were found for our pre-specified outcomes of anxiety, concern/worry and attendance at the clinic or hospital, and compliance to the intervention was generally high.

Increased MFA, as a finding of this review, might be attributed to an increasing maternal awareness with the use of FMC, and, consequently, women may feel reassured and relaxed as engaging with FMs provides a means of communicating with their baby or when they reach the recommended FM counts in a day^1,11,16^. The notion of psychological stress is associated with maternal psychological well-being^24^. Increased levels of anxiety, exposure to concern or worry affects the way pregnant woman cope with pregnancy and has shown to have neurological and cognitive developmental issues in infants^20,26,34^. Meta-analysis in this review showed no difference between the groups in levels of anxiety or concern/worry, which is reassuring in circumstances where formal FMC is used, or when women chose formal FMC as a means of engaging with their baby during pregnancy. In addition, mothers with a strong bond towards their fetuses have demonstrated lower levels of anxiety or worry^35^. Consequently, MFA levels were noted to increase, and anxiety/concern were reduced as women felt calm, safe and in control of their pregnancies^16,27^. In this sense, MFA and anxiety, concern or worry may be interlinked whereby FMC leads to higher MFA with no adverse psychological effects, or as others have shown higher levels of MFA correlate positively with lower levels of these stressors^35,36^. Moreover, MFA has been shown to have a protective effect against anxiety and improve a mother’s ability to cope with stress^37^. It is important to consider, however, that maternal concern could reflect general concern associated with term approaching or worry about labor, which may not be directly related to FMC^27^. Further research is required to explore the association between maternal anxiety, concern or worry and levels of MFA.

Concern has been expressed that introducing FMC as a routine method of screening FMs could increase the use of resources and admission rates, without influencing perinatal outcomes such as stillbirth and neonatal death^6,27,33^. Although no differences between the groups in hospital attendance or admission for concern regarding fetal activity or reduced FMs was found in this review, one needs to consider the variations and inconsistent definitions that exist globally in clinical practice with regard to FM assessment, as there is no standard protocol^5,18,38^, and many women do not receive adequate information on FMs or FMC methods^17,18^. Clinicians, when advising and instructing women about the significance of FMs in pregnancy, should ensure that the same standard of information is provided to all women. Evidence suggests that women value information and education that can be acquired from their clinicians, and as many as 84% of women have reported interest in FMC methods^6,18,39^. As the pattern and characteristics of FMs change closer to term, women should be informed of these changes as this information may help women familiarize themselves with their fetal activity and encourage them to seek immediate assistance, should there be a cause for concern. Future research focusing on how information regarding FM awareness and assessment is dispersed by healthcare providers is required.

### Strengths and limitations

The studies included in this review were largely of moderate or strong quality overall, providing reassurances for the strength of the evidence. Adequate data were available for a meta-analysis of our primary outcome, MFA, thus enhancing the representativeness of the result to the wider population. Limitations of the review, however, are also acknowledged. Women who were well educated and employed only were represented in some of the included studies, although categories of level of education or employment were not clearly defined^13,20,22^. Considering variables such as socioeconomic status and financial stability could possibly affect levels of pregnancy acceptance, MFA, and anxiety, worry or concern in a pregnant woman. The possibility of residual confounders and contamination across groups in the included large cluster trial should also be considered^27^. Lastly, a ‘Hawthorne effect’ was suggested by the authors of some of the included studies which potentially could have influenced the results of these studies^13,29^. Consequently, this effect may have transferred and influenced the overall results of our review by potentially leading to an over-estimate in the positive effect that formal FMC has on some subjective maternal psychological outcomes.

## CONCLUSIONS

Evidence suggests that FMC currently does not provide a benefit for reduced perinatal adversity, however, formal FMC as a means of assessing fetal well-being in pregnancy in some settings remains in use. In this context, this review has identified that FMC when used in clinical practice, does not adversely affect maternal psychological outcomes and can increase MFA. Although the studies in this review all used an FM chart for formal FMC, technological advances and accessibility are likely to advance the use of electronic or smart devices as replacements for FM charts in the future. This review provides informative findings that can assist clinicians in practice when discussing FM assessment in pregnancy with women, and the effect that formal FMC has on maternal subjective outcomes. Supporting women in their choice of FM assessment, including women who may choose to formally practice FMC, should be supported whereby women may favor or use FMC in communicating and bonding with their baby, and in becoming aware of their baby’s normal FM characteristics and patterns. Further research is required to establish the relationship between MFA and levels of anxiety in pregnant women.

## Data Availability

Data sharing is not applicable to this article as no new data were created.
